# Jatrorrhizine Balances the Gut Microbiota and Reverses Learning and Memory Deficits in APP/PS1 transgenic mice

**DOI:** 10.1038/s41598-019-56149-9

**Published:** 2019-12-20

**Authors:** Sheng Wang, Wei Jiang, Ting Ouyang, Xiu-Yin Shen, Fen Wang, Yu-hua Qu, Min Zhang, Tao Luo, Hua-Qiao Wang

**Affiliations:** 1 Department of Anatomy and Neurobiology, Zhongshan School of Medicine, Sun Yat-sen University, Guangzhou, Guangdong 510080 China; 2 Department of Anatomy, Histology and Developmental Biology, School of Basic Medical Sciences, Shenzhen University Health Science Centre, Shenzhen, 518060 China; 3 Pediatric Hematology and Oncology, Affiliated Guangzhou Women and Children’s Hospital, Zhongshan School of Medicine, Sun Yat-Sen University, Guangzhou, 510623 China

**Keywords:** Alzheimer's disease, Drug development, Molecular medicine

## Abstract

Alzheimer’s disease (AD) is a complex disorder influenced by both genetic and environmental components and has become a major public health issue throughout the world. Oxidative stress and inflammation play important roles in the evolution of those major pathological symptoms. Jatrorrhizine (JAT), a main component of a traditional Chinese herbal, coptidis rhizome, has been shown to have neuroprotective effects and we previously showed that it is also able to clear oxygen free radicals and reduce inflammatory responses. In this study, we demonstrated that JAT administration could alleviate the learning and memory deficits in AD. Furthermore, we also found that JAT treatment reduced the levels of Aβ plaques in the cortex and hippocampus of APP/PS1 double-transgenic mice. Other studies suggest that there are gut microbiome alterations in AD. In order to explore the underlying mechanisms between gut microbiota and AD, DNA sequencing for 16s rDNA V3-V4 was performed in fecal samples from APP/PS1 transgenic mice and C57BL/6 wild-type (WT) mice. Our results indicated that APP/PS1 mice showed less Operational Taxonomic Units (OTUs) abundance in gut microbiota than WT mice and with different composition. Furthermore, JAT treatment enriched OTUs abundance and alpha diversity in APP/PS1 mice compared to WT mice. High dose of JAT treatment altered the abundance of some specific gut microbiota such as the most predominant phylum Firmicutes and Bacteroidetes in APP/PS1 mice. In conclusion, APP/PS1 mice display gut dysbiosis, and JAT treatment not only improved the memory deficits, but also regulated the abundance of the microbiota. This may provide a therapeutic way to balance the gut dysbiosis in AD patients.

## Introduction

Alzheimer’s disease (AD) is characterized by progressive impairment of cognition and memory as well as pathological hallmarks which include extracellular amyloid plaques, intracellular neurofibrillary tangles and neuronal loss.AD mainly affects the elderly who undergo slow but progressive loss of memory and cognitive functions. The pathological changes of AD include atrophy of the cortex, widening of the sulci, enlargement of the ventricles and substantial reduction of neurons. These pathological changes are characterized by significant accumulation of proteinaceous fibrillary substances and neurofibrillary tangles as well as reduction of choline acetylcholine and acetylcholine^1,2^. AD has been studied extensively for decades, however, the disease pathogenesis still remains unclear and there has been no effective treatment discovered yet. Previous AD research mainly focusses on Aβ production and degradation, tau abnormal phosphorylation, and other brain-related aspects. Recently, intestinal microbiome has been shown to be one of the emerging risk of AD progression^3^.

In humans, only half of the cells and a tiny number of genes are unique, and the rest can be found in gut microbiota such as viruses, archaea and fungi. There has been a rise in the understanding of human diseases and the emerging medical prospects of gut microbiome^4–6^. Gut microbiota has been shown to contribute to many complex central nervous system disorders^3,7^. For instance, alterations of gut microbiota have been identified in many neurodegenerative diseases such as Parkinson’s disease^8,9^, multiple sclerosis^10^, autism disorder^11^ and AD^12,13^. Phyla or strains of bacteria in feces of 5xFAD Alzheimer’s mice Model are distinguishable from their wild type littermate^14^. Microbial colonies of AD patients have been characterized compositionally different from that of control individuals^3^. Furthermore, correlation analyses of gut microbiota and blood cytokines suggest that gut microbiota may promote brain inflammation. Some studies reported that gut-microbiota-related dysbiosis will enhance inflammation and gut’s leakiness^15^, and through a web of interactions, it increases neuronal dysfunction and apoptosis, at the same time, promotes learning and memory impairment^16,17^. Other studies suggest that there are gut microbiome alterations in AD, and learning and memory impairment was related to gut microbiota disorder in aging mice^18^. Studies by Harach *et al*.^19^, further revealed that the germ-free APP transgenic mice showed a significant decrease in cerebral Aβ levels compared with control mice.

With the progression of research on Alzheimer’s disease, different drugs have been extensively investigated. However, most of them just improve the severity of disease symptoms without finding successfully the fundamental causes^20^. Therefore, it is crucial to find drug candidates targeting the prevention of neural loss as well as the alleviation of Aβ aggregation^21^. Jatrorrhizine (JAT) is a tetrahydro-isoquinoline alkaloid isolated from a traditional Chinese medicine Huang Lian that is capable of sterilization and often used as a detoxification and anti-hyperglycemic agent. A number of studies confirmed that JAT has multiple biological functions such as anti-oxidation^22^. Our previous studies indicated JAT can be used to protect neurons against hydrogen peroxide or Aβ oligomer induced cell damage^23^. JAT has potential to clear oxygen free radicals and to reduce the inflammatory responses on neurons. We also demonstrated that JAT can alleviate the impairment of learning and memory abilities in APP/PS1 mice in this study. Donepezil hydrochloride, as an acetylcholinesterase inhibitor, is a long-acting drug to treat symptoms of AD^24^. Here, we used it as a positive control to evaluate the effect of JAT on AD.

## Materials and Methods

### Reagents and antibodies

JAT (C_20_H_20_NO_4_, FW 338.38, purity > 98%) was obtained from Chengdu Herbpurify co., LTD (Chengdu, Sichuan, China). Poly (ethylene glycol) average Mn300 (H(OCH_2_CH_2_)nOH) and DONE (C_24_H_29_NO_3_ · HCl · H_2_O, FW 433.97, purity >98%) were purchased from Sigma-Aldrich (Saint Louis, Mo, USA). Physiological saline for i.p. injection was bought from Guangzhou Cisen medical Science and Technology Co., LTD (Guangzhou, Guangdong, China). JAT was dissolved in poly (ethylene glycol) and diluted with saline. Antibodies used for immunostaining were anti-amyloid-β (clone 6E10, Catalog SIG-39320, Covance), anti-GFAP polyclonal antibody (Catalog AB5541, Millipore Corporation), Cy5 (Jackson Immunoresearch, USA), Alexa Fluor 488 (Jackson Immunoresearch, USA).

### Animals and drug administration

APP/PS1 transgenic mice (male, APP/PS1) and wild-type (male, C57BL/6) mice were purchased from Guangdong Medical Laboratory Animal Research Institute. Twenty-four APP/PS1 mice were randomly allocated to receive high dose JAT (JATH, n = 6), low dose JAT (JATL, n = 6), saline (APP/PS1, n = 6), donepezil hydrochloride monohydrate (DONE, n = 6) for 24 weeks at an age of 3 months. Six age-matched C57BL/6 mice were also being given saline for 24 weeks via intraperitoneal injection. The mice were housed in an air-conditional room (temperature 19 °C~23 °C), under a circadian rhythm ensuring night time of more than 12 hours (lights on from 07:00 am to 07:00 pm) with free access to sterile water and food. The animal experiments were approved by the Institutional Animal Care and Use Committee(IACUC), Sun Yat-Sen University, and all animal care and procedures conform to the National Institutes of Health Guide for Care and Use of Laboratory Animals. JATH and JATL were intraperitoneally administered at the doses of 10 mg/kg/day and 5 mg/kg/day, respectively, for 6 months. Mice in group DONE were treated with DONE at the dose of 0.3 mg/kg/day through intraperitoneal injection. Age-matched mice in group APP/PS1 and C57BL/6 were injected with equal volume of physiological saline.

### Morris water maze test

To assess the spatial learning and memory abilities of APP/PS1 mice and WT mice, Morris water maze test was performed in a water tank with a diameter of 80 cm which was filled with water (19 °C~23 °C). The tank was divided into four equidistant quadrants. Titanium dioxide was added to keep water opaque, and water was added until it was 1 cm over the platform. The procedure included acquisition trail and probe trial sections. From day 1 to day 5, mice were devoted into the tank in one of the four quadrants to find the hidden-platform, the escape latency (time taken to find the platform) was recorded using a computer-controlled video tracking system to analyze the spatial learning ability. The mice that failed to find the platform within 60 s were manually guided to the platform for several minutes. Probe trial was carried out on day 6, the platform was removed, and the mice was allowed to swim for 60 s, and the time spent in the target quadrant and number of times that the mice crossed the position where the platform used to be located were recorded and analyzed to assess the spatial memory(Vorhees and Williams, 2006).

### Immunostaining

After behavioural test, mice were anesthetized with 2% urethane and perfused with 0.9% NaCl. The brains of three wild-type and twelve APP/PS1 mice were removed and fixed in 4% paraformaldehyde (in PBS) at 4 °C for a week. After dehydration in 30% sucrose (in PBS), 30-μm-thick slices were cut using a Cryostat (CryoStar NX50, Thermo Scientific, USA). For general immunostaining, sections were pre-treated by formic acid (70%) for 10~30 minutes at room temperature, and then washed in PBS for three times and blocked in 5% normal goat serum supplemented with 0.1% Triton X-100 (in PBS) at room temperature for 1 h. Primary antibodies (anti-Aβ monoclonal antibody, Catalog SIG-39320, Covance, USA; anti-GFAP polyclonal antibody, Catalog AB5541, Millipore Corporation) were incubated at 4 °C overnight. On the second day, the sections were washed thrice in PBS for 3 times and corresponding species-derived fluorophore (Cy5, Jackson Immunoresearch, USA; Alexa Fluor 488, Jackson Immunoresearch, USA)-conjugated secondary antibodies were added at room temperature and incubated for 1 h. Hoechst 33342 (Sigma-Aldrich) was used for the visualization of nuclei. After another three rounds of washes in PBS, images of hippocampal region along the AP axis (Bregma −1.5 ~ −2.5 mm) were mounted and imaged using fluorescent microscope (BX63, Olympus, Japan).

### Quantification of Aβ plaques and reactive astrocytes

The Aβ plaques and reactive astrocytes were quantified by a blinded observer using ImageJ in three sections per brain (Bregma, −1.5 mm to −2.5 mm) in 3 animals per group. We chose ten appropriate fields in each slice and calculated the arithmetic means of each region. Among all parameters, cells at the edge of the tissue sections were not considered for evaluation to avoid human error statistics maximally. We manually adjusted the analyzed areas so that only the area of interest was accurately measured with each measurement, and adjusted to exclude brains regions other than the cortex and hippocampus. The area of Aβ plaques in the cross section of the brain was recorded, as well as the total number of Aβ plaques determined by immunostaining. To quantify reactive astrocytes, the percentage of threshold pixels per image to the total pixels in the region of interest was calculated and presented as the percentage of affected tissue.

### 16S rDNA amplicon sequencing of gut microbiota

After administrating JAT, DONE or saline for 6 consecutive months (before the Morris water maze test), the feces were collected at successive a few mornings and frozen to ultra-low temperature refrigerator until use. Total genomic DNA (gDNA) of fecal samples was isolated using PowerSoil-htp 96 Well Soil DNA Isolation Kit (12955, Mobio, USA) according to the manufacturer’s protocol. Universal primers employed to amplify V3-V4 regions of bacterial 16S rDNA genes from gDNA were 5′-ACTCCTACGGGAGGCAGCA-3′ and 5′-GGACTACHVGGGTWTCTAAT-3′. The libraries were sequenced on Illumina Hiseq platform (Illumina, San Diego, CA, USA) to generate 2 × 250-bp pair-end sequencing reads according to the standard protocol provided by Illumina.

### Sequence analysis

PE reads were spliced through the overlap by using FLASH v1.2.7 software, then the original Tags data (Raw Tags) was obtained. High quality Tags were generated by filtering the raw tags in Trimmomatic v0.33 software. We used UCHIME v4.2 software to identify and remove chimeras Sequence, and to get the final effective data (Effective Tags). Operational taxonomic unit (OTU) was clustered and taxonomic annotation were performed in QIIME (version 1.8.0)^25^. The abundance of species at different levels (phylum, class, order, family, genus, and species) of classification was also by performing the QIIME software (version 1.8.0). Mothur (version v.1.30) was used to generate alpha diversity indices (observed OTU, ACE index, Chao1 index, Simpson index, Shannon index and Goods coverage)^26^. QIIME (version 1.8.0) was also used to measure beta diversity indices (unweighted UniFrac distance and weighted UniFrac distance)^27^. Line Discriminant Analysis (LDA) Effect Size (Lefse) analysis was used for screening significantly different biomarkers^28^. To study the differences in the abundance of microbial colonies between the two groups of samples, the species abundance data between groups were tested by Metastats^29^. PICRUSt software relates the functional genes in the sample by comparing the species composition information obtained from the 16S sequencing data to analyze the functional differences between the different samples or groups^30^. Correlation analysis was performed according to Sparcc algorithm^31^.

### Statistical analysis

Date analyses were performed with SPSS 23.0. Data obtained from acquisition trail were analyzed by repeated measures and multivariate analysis of variance (ANOVA) process of the genera linear model in SPSS, post hoc comparisons were assessed using the LSD test or Dunnett’s T3 test. The other data were presented as the mean with the standard error of mean (SEM). Verifying that the mean values of multiple groups was performed using a one-way analysis of variance test (ANOVA) and the differences between groups were compared with the least significant difference test. Results were considered statistically significant when p < 0.05. GraphPad Prism (version 7) was used to conduct statistical tests and graphs.

### Ethics approval and consent to participate

The animal experiments were approved by the Animal Ethical and Welfare Committee of SYSU, and all animal care and procedures conform to the National Institutes of Health Guide for Care and Use of Laboratory Animals.

## Results

### JAT ameliorated learning and memory deficits in APP/PS1 transgenic mice

**Figure 1:**
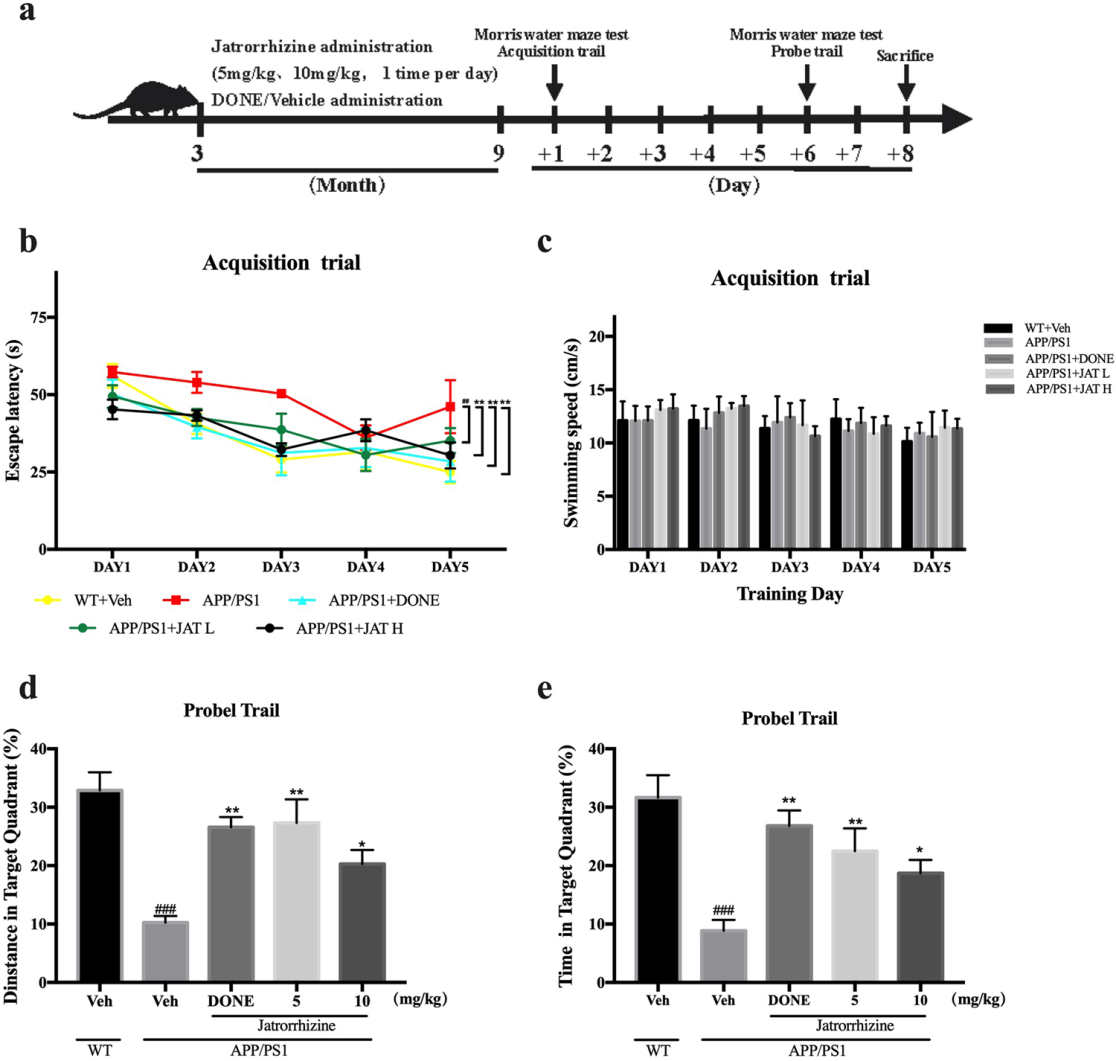
JAT ameliorated learning and memory deficits in APP/PS1 transgenic mice. (**a**) Schedule of behavioral test. Three-month-old male APP/PS1 mice were intravenously injected with different concentrations of Jatrorrhizine (5 mg/kg, 10 mg/kg, n = 6*2), DONE (0.3 mg/kg/day, n = 6) and vehicle (2.5% DMSO in PBS, n = 6) for 6 months (1 time per day). As controls, age-matched male wild-type (Wt) mice were injected with vehicle (2.5% DMSO in PBS, n = 6) for 6 months. (**b**) Jatrorrhizine administration groups have shown shorter escape latencies to the hidden platform in comparison to the vehicle-treated APP/PS1 mice. (**c**) During the acquisition trial, there are no significant differences in the swimming speed of mice. In the probe trial, APP/PS1 mice treated with Jatrorrhizine showed more exploratory distance (**d**) and time (**e**) in the target quadrant compared with the vehicle-treated APP/PS1 mice. Data information: The data are expressed as mean ± SEM. For statistical significance, ^###^P < 0.001 compared to WT mice, *P < 0.05, **P < 0.01, ***P < 0.001 compared to vehicle-treated APP/PS1 mice.

We injected different concentrations of JAT intraperitoneally to three months old male APP/PS1 mice for 6 months, while the age-matched male wild-type (WT) control received the vehicle (n = 6) (Fig. 1a). We subjected all these mice to a Morris water maze test 2 days after the last administration of JAT to assess alterations in learning and memory abilities.

The Morris water maze assay was used to measure the spatial learning and memory in APP/PS1 mice. During the acquisition trail, the mice showed a gradual reduction in the time required to locate the platform (Fig. 1b). Repeated measures ANOVA indicated that there were significant differences in latent phases between all experimental groups. The Dunnett’s multiple comparisons test suggested that WT and DONE group exhibited a shorter escape latency than vehicle-treated APP/PS1 mice at days 5 (P < 0.01) of acquisition trails. It was noteworthy that the swimming speed of mice did not show any statistically significant difference among all groups (P > 0.05). This result indicated that the differences in escape latencies were not due to the travel rate of mice (Fig. 1c). About 24 hours after the last acquisition training, hidden platform was removed from the target quadrant and the mice were tested for spatial memory in the probe trials. As shown in Fig. 1d,e, analysis by one-way ANOVA displayed remarkable differences among each group (P < 0.01). The Dunnett’s multiple comparisons test revealed that compared to WT mice, APP/PS1 mice treated with vehicle spent the minimum time exploring and also moved the least within the target quadrant. However, these behavioral deficits were reversed by 6 months long JAT administration in APP/PS1 transgenic mice. JAT has a marked impact on both two dose groups, which significantly increased the time spent in the target quadrant by APP/PS1 mice. In addition, JAT also obviously increased exploratory distance the APP/PS1 mice moved in the platform quadrant.

### JAT significantly reduces Aβ plaques in the cortex and hippocampus of APP/PS1 mice

Pathogenesis of AD involves Aβ aggregating into insoluble clusters or plaques in the brain^32^, and this formation of these plaques contributes to the learning and memory deficits^33^. Given that pretreatment with different concentrations of JAT can obviously inhibit neurotoxicity, we hypothesized that JAT might act on Aβ aggregates directly to alleviate the neurotoxicity in AD.

**Figure 2:**
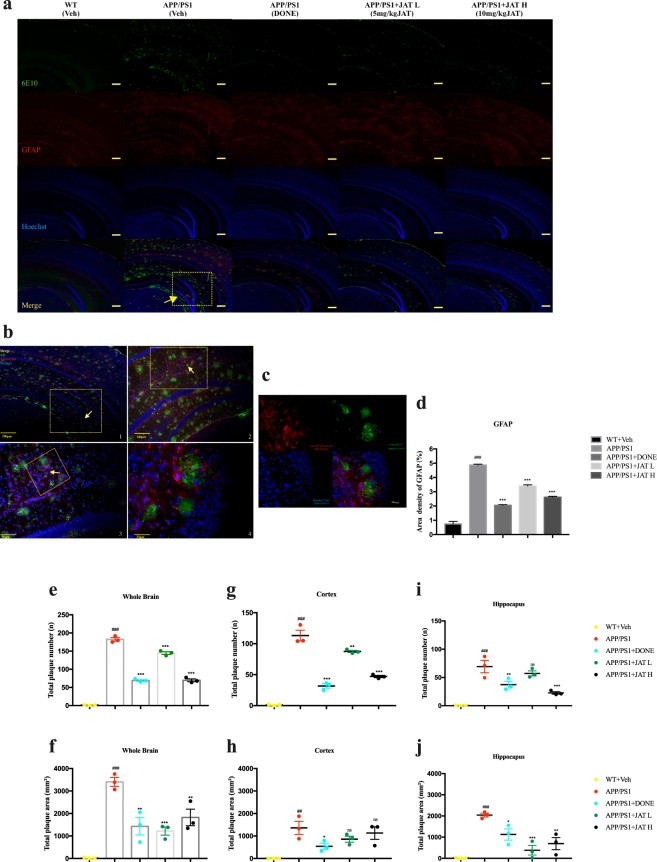
Jatrorrhizine reduces Aβ plaques in the brains of APP/PS1 mice. (**a**) Immunohistochemical analysis of cortical and hippocampal regions of the brains in wild-type (Wt) and APP/PS1 mice after administration. Diffuse plaques in the brain sections were stained by anti-Aβ antibody (clone 6E10, green colour) and anti-GFAP antibody (red colour). Hoechst 33342 (blue colour) was applied for nuclear counterstaining. Scale bars = 200 μm. (**b**) Magnification of one segment of the immunostaining in APP/PS1 group from (**b**) (Scale bars = 200 μm, 100 μm, 50 μm, 20 μm, clockwise respectively). As shown in (**c**), diffuse plaques co-localized with reactive astrocytes in APP/PS1 mouse brains. (**d**) Statistics of GFAP in the brains of each group. (**e–j**) Statistics of Aβ plaques in the brains of each group. Total numbers and areas of Aβ plaques in the whole brains (**e,f**), cortex (**g,h**) and hippocampus (**i,j**). Data information: In (**e–j**), data are presented as mean SEM. ^##^P < 0.01, ^###^P < 0.001 compared to WT mice, *P ≤ 0.05, **P ≤ 0.01 and ***P ≤ 0.001 (one-way ANOVA followed by Bonferroni’s post hoc comparisons tests).

To assess the effect of JAT on plaque formation in the brain, we evaluated the levels of Aβ plaques in mice that underwent behavioral tests. Evidence from previous studies have suggested that diffuse Aβ plaques in AD are relevant to reactive astrocytes that converge around them^34^ leading to neurodegenerative events^35^. Hence, in order to detect reactive astrocytes and diffuse plaques in the brains of mice, we performed immunostaining respectively, using anti-glial fibrillary acidic protein (GFAP) antibody and 6E10 antibody. We clearly observed the co-localization of reactive astrocytes and diffuse plaques in APP/PS1 mice brains (Fig. 2b,c). In addition, our results further showed that JAT administration had a reduced expression of diffuse plaques and GFAP in the hippocampus and cortex of APP/PS1 transgenic mice (Fig. 2a,d). Moreover, overall numbers and areas of plaques were significantly decreased in whole brains of JAT-administered APP/PS1 mice compared with the level in vehicle treatment groups (Fig. 2e,f). This tendency was observed not only in the cortex (Fig. 2g,h), but also in hippocampus (Fig. 2i,j). The above data clearly demonstrated that JAT administration decreased Aβ plaques in different brain regions of APP/PS1 mice.

### APP/PS1 transgenic mice showed a remarkably different composition of gut microbiota compared to C57BL/6 mice

#### APP/PS1 mice had lower OTUs abundance and alpha diversity compared to C57BL/6 mice

**Figure 3:**
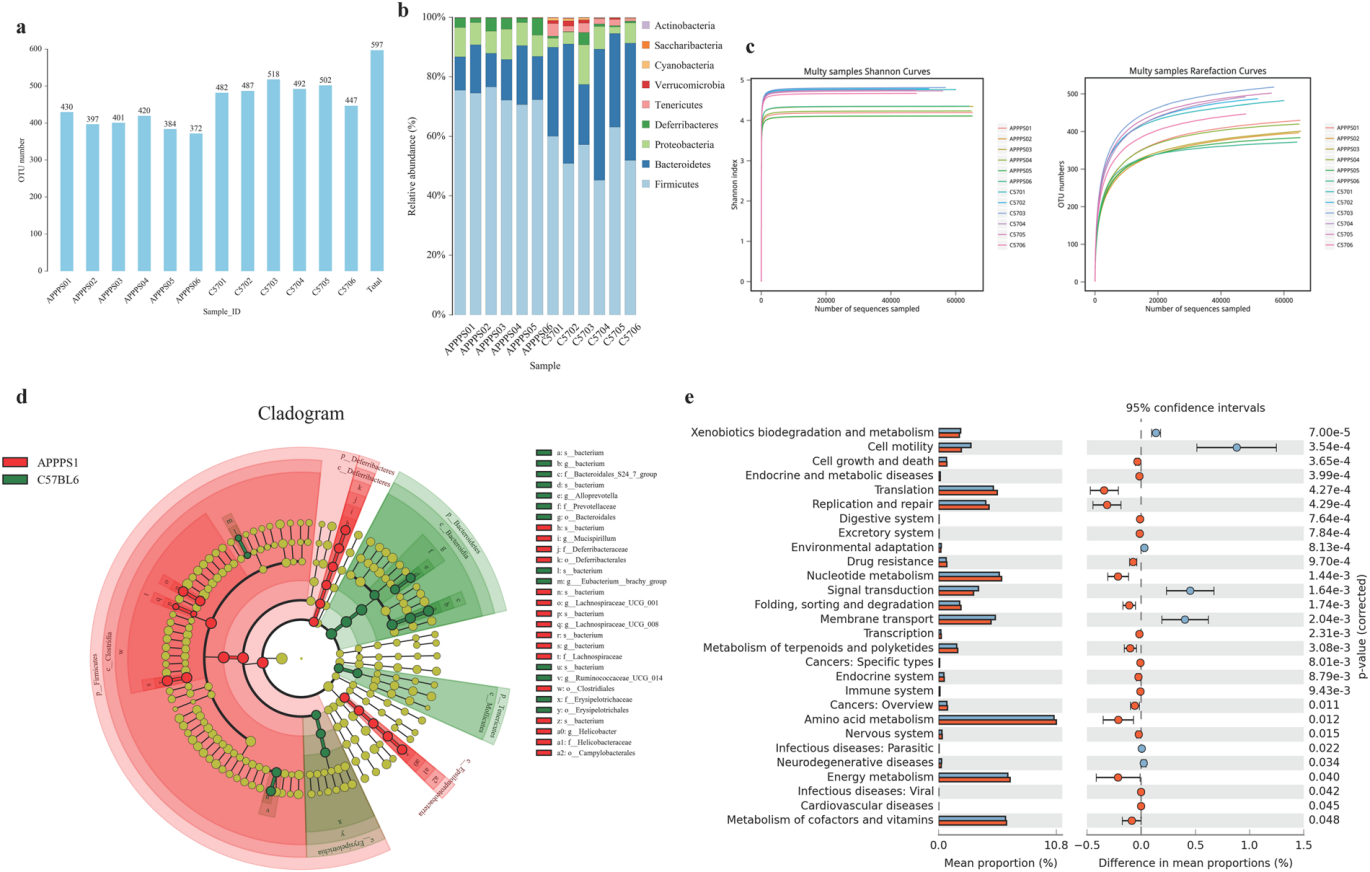
APP/PS1 transgenic mice showed a remarkably different composition of gut microbiota compared to C57BL/6 mice. We sequenced the fecal samples from 6 APP/PS1 and 6 C57BL/6 mice. (**a**) Amounts of operational taxonomic units (OTUs) in APP/PS1 mice and C57BL/6 mice. (**b**) Alpha diversity in C57BL/6 mice and APP/PS1 mice. *(NOTE: The abscissa of this image is sample’s name and the ordinate is the relative abundance percentage. Each color represents a species, and the area of each color represents the relative abundance ratio)*. (**c**) The identity of 97% of OTUs between APP/PS1 and C57BL/6 were measured, and (**d**) showed the amounts of phylum Firmicutes, phylum Bacteroidetes and Tenericutes in APP/PS1 mice and C57BL/6 mice. (**e**) Predictive assessment of the microbial community functional potential (PICRUst) was performed to record differences and changes in the metabolic pathways between APP/PS1 mice and C57BL/6 mice.

To assess the potential correlations between AD and gut microbiota, we sequenced the fecal samples from 6 APP/PS1 and 6 C57BL/6 mice. In total 851,851 raw tags (743,120 clean tags) were obtained from 12 samples with an average of 61927 ± 7037 clean tags. We found the number of operational taxonomic units (OTUs) in APP/PS1 mice was significantly lower (p < 0.001) than that of C57BL/6 mice (Figs. 3a, S1a), indicating a lower gut microbiota abundance in APP/PS1 mice. Alpha diversity reflects the diversity of species within a single sample, with a variety of metrics: Chao1, Ace, Shannon, and Simpson. Alpha diversity was significantly higher in C57BL/6 mice compared to APP/PS1 mice (observed Chao1 index, *p* < *0.01*; Ace index, *p* < *0.001*; Shannon index, *p* < *0.001*) (Figs. 3b, S1b–e). Many studies have pointed out that the high diversity of bacterial flora usually indicates better health status of the host. Analysis of beta diversity using principal coordinate’s analysis (PCoA) and Non-MetricMulti-Dimensional Scaling (NMDS) indicated that the microbiota composition was dramatically different between APP/PS1 and C57BL/6 mice. Figure 5c,d shows tightly grouped microbiota composition in C57BL/6 mice, while much more scattered in APP/PS1 mice.

**Figure 4:**
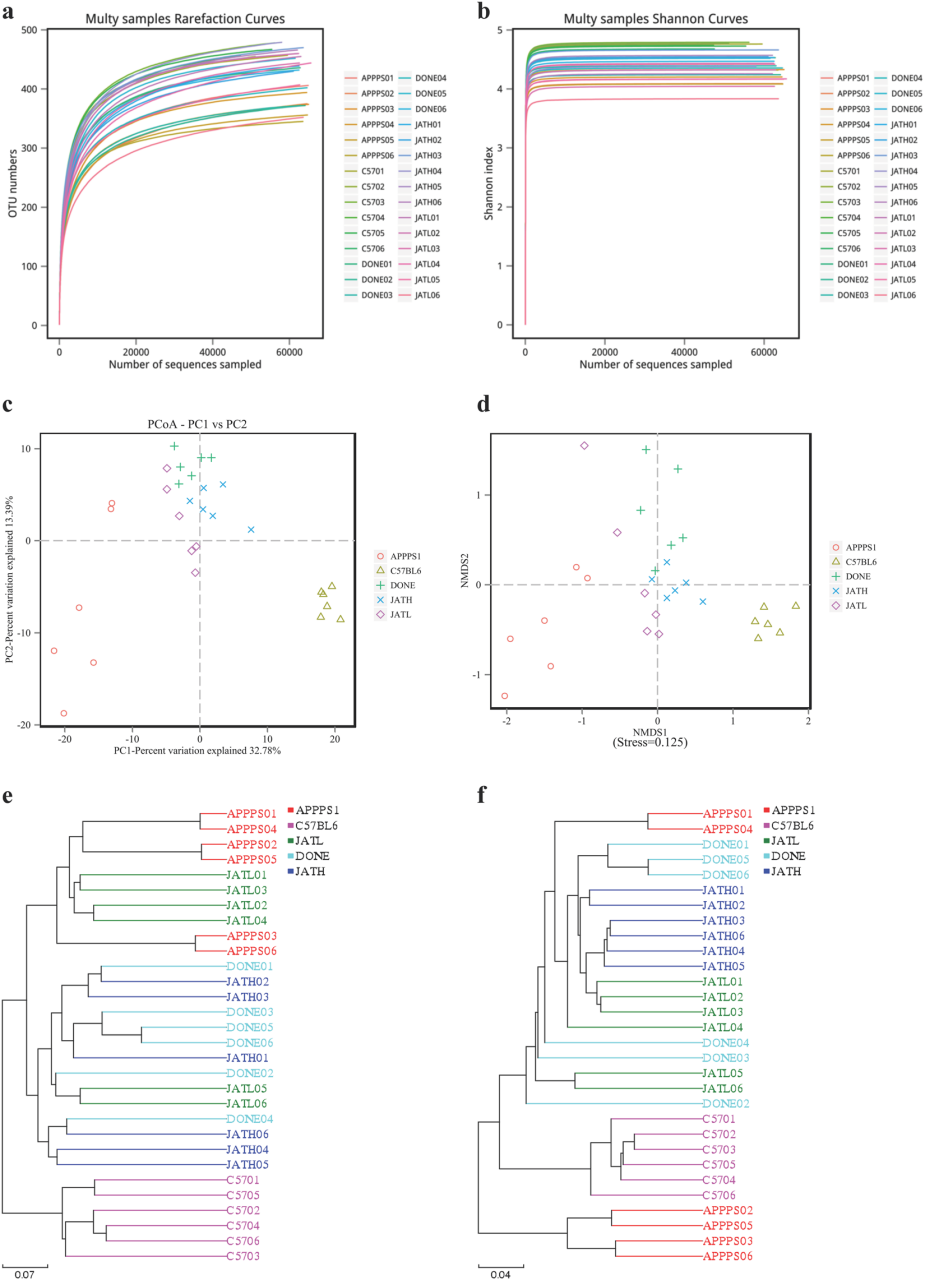
The JAT treatment modulated the imbalance of gut microbiota in APP/PS1 transgenic mice. We collected fecal samples of APP/PS1 mice from group JATH, JATL and DONE, and then performed 16S rDNA amplicon sequence. Mean OTU numbers (**a**) and alpha diversities (**b**) of each experimental group were measured. Principal coordinate’s analysis (PCoA) (**c**) and Non-MetricMulti-Dimensional Scaling (NMDS) (**d**) were used to make analysis of beta diversity and showed the microbiota composition among groups. (**e,f**) Unweighted pair-group method with arithmetic mean (UPGMA) based on OTU or phylogenetic was used to show the microbiota of mice feces.

**Figure 5:**
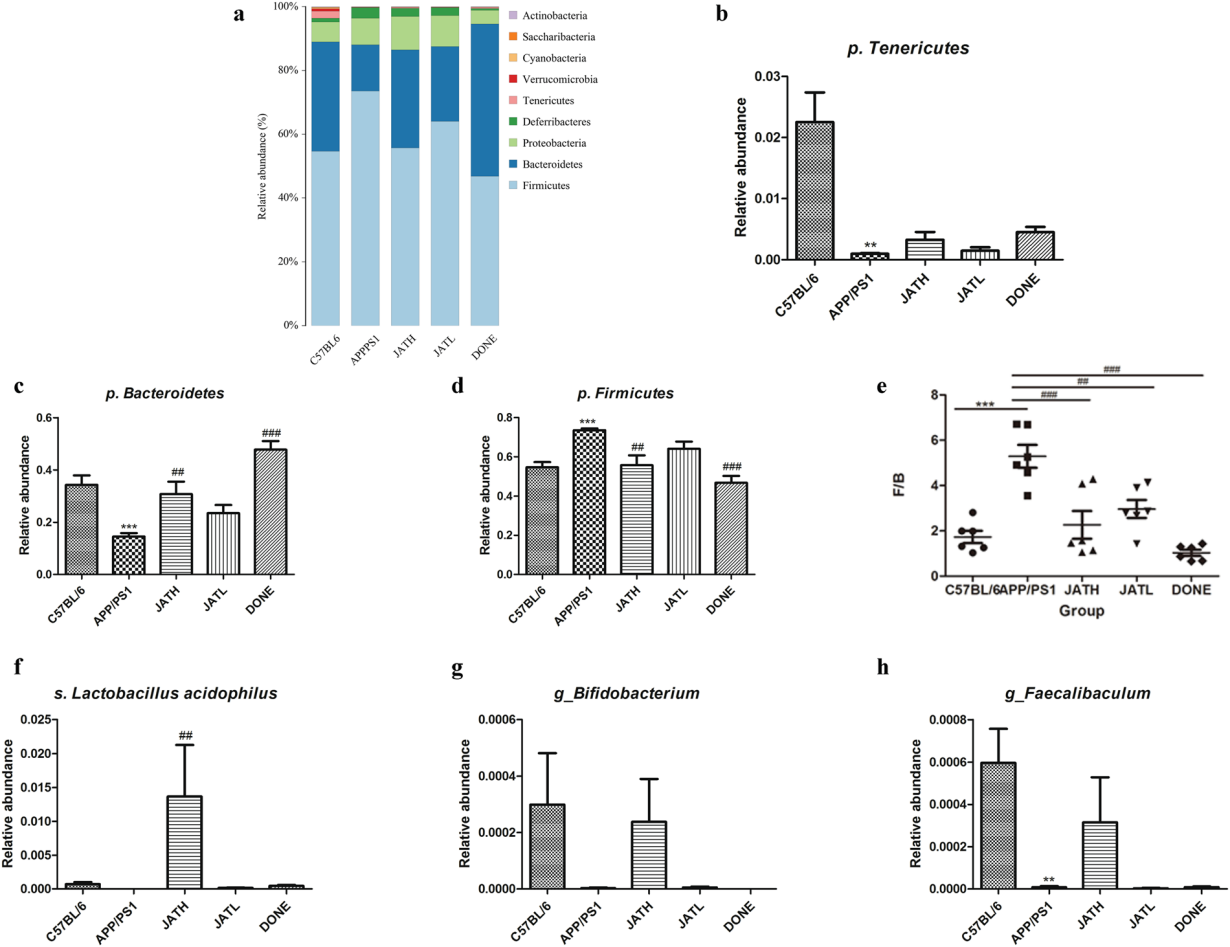
The JAT treatment modulated the imbalance of gut microbiota in APP/PS1 transgenic mice. We collected fecal samples of APP/PS1 mice from group JATH, JATL and DONE, and then performed 16S rDNA amplicon sequence. (**a**) Relative abundance (%) of species within a single sample of all experimental groups. (**b–h**) Statistics in the abundance of some bacteria. Amounts of Phylum Tenericutes (**b**), Bacteroidetes (**c**), phyla Firmicute (**d**), the ratio of Firmicutes and Bacteroidetes (F/B) (**e**), Lactobacillus acidophilus (**f**), Bifidobacterium (**g**) and Faecalibaculum (**h**).

#### Abundance of certain bacteria is severely changed in APP/PS1 mice

The identity of 97% of OTUs was distributed across 9 phyla and there were distinguishable changes between APP/PS1 and C57BL/6 (Fig. 3c). The amount of phylum *Firmicutes*, which made up the predominant bacterial division, increased and the amount of phylum *Bacteroidetes* and *Tenericutes* dramatically decreased in APP/PS1 mice compare to C57BL/6 mice (Fig. 3d). In this study, we performed the abundance analyses between the two groups at the level of phylum, class, order, family, genus and species. At a false discovery rate of 5%, we identified 89 differentially abundant taxa within the phylum, class, order, family and genus levels. APP/PS1 transgenic mice also had different abundance of certain gut microbiota compared to C57BL/6 as shown in Table 1. Several taxa were only detected in C57BL/6 mice, but not in APP/PS1 mice and these included phylum *Verrucomicrobiae*, family *Peptostreptococcaceae*, genus *Akkermansia* and *Allobaculum*, indicating C57BL/6 mice had an abundance fecal microbiota (Table 1). *Peptostreptococcaceae* is a beneficial gram-positive bacterium that can synthesize short-chain fatty acids in the intestine that can play an anti-inflammatory role by promoting the production of indole acrylic acid^36^. In addition, *Akkermansia* is an important probiotic that can help alleviate obesity and lower blood glucose level^37^. The remarkable difference between APP/PS1 and C57BL/6 mice indicated APP/PS1 had a dysbiosis.

**Table 1:** Abundance of certain bacteria is severely changed in APP/PS1 mice.

	P value	Mean(C57BL6)	Mean(APP/PS1)	Log2 fold change
**Phylum**				
Firmicutes	0.000000	0.548000	0.737000	−0.43
Bacteroidetes	0.000200	0.342000	0.145000	1.24
Tenericutes	0.001800	0.023100	0.000968	4.58
Actinobacteria	0.013600	0.000984	0.000082	3.58
Cyanobacteria	0.013900	0.004410	0.000208	4.41
Verrucomicrobia	0.018700	0.007350	ND.	
Deferribacteres	0.039600	0.011600	0.033200	−1.52
**Class**				
Clostridia	0.000000	0.508000	0.731000	−0.53
Betaproteobacteria	0.000353	0.007050	0.000005	10.43
Bacteroidia	0.000706	0.342000	0.145000	1.24
Mollicutes	0.001290	0.023100	0.000968	4.58
Coriobacteriia	0.001650	0.000685	0.000080	3.11
Erysipelotrichia	0.002590	0.027100	0.000485	5.80
Epsilonproteobacteria	0.010700	0.019000	0.051600	−1.44
Melainabacteria	0.013700	0.004410	0.000208	4.41
Alphaproteobacteria	0.017000	0.003890	0.000028	7.11
Verrucomicrobiae	0.018700	0.007350	ND.	
Deferribacteres	0.038300	0.011600	0.033200	−1.52
**Order**				
Bacteroidales	0.000000	0.342000	0.145000	1.24
Burkholderiales	0.000000	0.007050	0.000005	10.43
Clostridiales	0.000000	0.508000	0.731000	−0.53
Mollicutes_RF9	0.000000	0.015300	0.000730	4.39
Coriobacteriales	0.000632	0.000685	0.000080	3.11
Erysipelotrichales	0.001110	0.027100	0.000485	5.80
Campylobacterales	0.010200	0.019000	0.051600	−1.44
Gastranaerophilales	0.012500	0.004410	0.000208	4.41
Rhodospirillales	0.015600	0.003890	0.000028	7.11
Verrucomicrobiales	0.016700	0.007350	ND.	
Anaeroplasmatales	0.030900	0.007610	0.000003	11.54
Deferribacterales	0.036900	0.011600	0.033200	−1.52
**Family**				
Lachnospiraceae	0.000000	0.342000	0.583000	−0.77
Alcaligenaceae	0.000355	0.007050	0.000005	10.43
Christensenellaceae	0.000839	0.000510	0.000008	6.05
Coriobacteriaceae	0.001100	0.000685	0.000080	3.11
bacterium	0.001260	0.019700	0.000938	4.39
Bacteroidales_S24-7_group	0.001740	0.176000	0.008280	4.41
Family_XIII	0.001900	0.001650	0.000557	1.57
Prevotellaceae	0.002350	0.031900	0.000839	5.25
Erysipelotrichaceae	0.002520	0.027100	0.000485	5.80
Peptostreptococcaceae	0.007900	0.001180	ND.	
Helicobacteraceae	0.011200	0.019000	0.051600	−1.44
Rhodospirillaceae	0.019900	0.003890	0.000028	7.11
Rs-E47_termite_group	0.020100	0.007930	0.003740	1.08
Verrucomicrobiaceae	0.021200	0.007350	ND.	
Anaeroplasmataceae	0.033400	0.007610	0.000003	11.54
Deferribacteraceae	0.037900	0.011600	0.033200	−1.52
**Genus**				
Tyzzerella_3	0.000013	0.000010	0.003250	−8.35
Parasutterella	0.000158	0.007050	0.000005	10.43
Ruminococcaceae_NK4A214_group	0.000303	0.000921	0.000442	1.06
Intestinimonas	0.000421	0.000190	0.000640	−1.75
Parabacteroides	0.000513	0.005240	0.000208	4.65
Ruminococcaceae_UCG-010	0.000632	0.001060	0.000023	5.52
Rikenellaceae_RC9_gut_group	0.000737	0.009720	0.002680	1.86
Lachnospiraceae_UCG-001	0.000829	0.013700	0.058000	−2.08
Coriobacteriaceae_UCG-002	0.001550	0.000349	ND.	
Enterorhabdus	0.001640	0.000335	0.000080	2.07
Alloprevotella	0.001960	0.025900	0.000495	5.71
Rikenella	0.002750	0.002670	0.007560	−1.50
Faecalibaculum	0.002870	0.000601	0.000008	6.29
Christensenellaceae_R-7_group	0.002960	0.000332	0.000008	5.43
Ruminococcaceae_UCG-014	0.003070	0.048400	0.008800	2.46
[Eubacterium]_brachy_group	0.003550	0.000162	0.000044	1.89
Anaerotruncus	0.004510	0.008920	0.019400	−1.12
Prevotellaceae_UCG-001	0.004640	0.002800	0.000323	3.12
Family_XIII_AD3011_group	0.004920	0.000149	ND.	
Acetitomaculum	0.005040	0.000253	0.001670	−2.72
Ruminococcaceae_UCG-003	0.005880	0.000760	0.000082	3.21
Ruminococcaceae_UCG-005	0.007540	0.000616	0.000072	3.09
Lachnospiraceae_UCG-008	0.007840	0.000010	0.000098	−3.33
Helicobacter	0.008090	0.019000	0.051600	−1.44
Roseburia	0.011500	0.011000	0.022500	−1.03
Lachnospiraceae_FCS020_group	0.012400	0.001510	0.000347	2.12
Ruminiclostridium	0.013700	0.007910	0.016200	−1.03
Turicibacter	0.013800	0.013800	0.000049	8.15
Tyzzerella	0.014800	0.004590	0.000993	2.21
Akkermansia	0.016800	0.007350	ND.	
[Eubacterium]_xylanophilum_group	0.017000	0.011800	0.002470	2.26
Ruminococcus_1	0.018000	0.003710	0.000093	5.32
Allobaculum	0.025600	0.000922	ND.	
Anaeroplasma	0.026100	0.007610	0.000003	11.54
[Eubacterium]_nodatum_group	0.028300	0.000721	0.000054	3.74
Mucispirillum	0.030800	0.011600	0.033200	−1.52
Ruminococcaceae_UCG-013	0.035100	0.002350	0.000062	5.25
bacterium	0.039200	0.354000	0.278000	0.35
Lachnospiraceae_NK4A136_group	0.042100	0.164000	0.228000	−0.48
Lachnospiraceae_UCG-010	0.042100	0.000073	0.000218	−1.58
Acetatifactor	0.044900	0.000423	0.002780	−2.72
Prevotellaceae_NK3B31_group	0.047400	0.002020	ND,	
Candidatus_Stoquefichus	0.047800	0.001140	0.000074	3.94
**Species**				
rumen_bacterium	0.009250	0.009670	ND.	
bacterium	0.016500	0.964000	0.980000	−0.02

Note: ND. NOT Detected.

#### Functional analysis of APP/PS1 mice and C57BL/6 mice

We used a predictive assessment of the microbial community functional potential (PICRUst), and then compared different abundances of KOs through STAMP to study differences and changes in the metabolic pathways between APP/PS1 mice and C57BL/6 mice. C57BL/6 mice had more genes involved in the metabolic pathways, particularly in endocrine and metabolic disease, translation and drug resistance (Welch’s t-test, *p* < *0.001*). However, genes associated with cell motility, environment adaptation, xenobiotics biodegradation and metabolism, were more abundance in APP/PS1 (Welch’s t-test, *p* < *0.001*) (Fig. 3e).

### The JAT treatment modulated the imbalance of gut microbiota in APP/PS1 transgenic mice

#### JAT administration improved the OTUs abundance and alpha diversity of APP/PS1 mice

Donepezil is a clinical drug inhibited the activity of acetylcholinesterase and butyrylcholinesterase, thus enhancing cognitive function^38^. Here, donepezil was used as a positive control, to evaluate the anti-aging effect and regulating effect on intestinal microbiota. We collected fecal samples of APP/PS1 mice from group JATH, JATL and DONE, and then performed 16S rDNA amplicon sequence.

When compared to mice in saline administered control APP/PS1 group, the high dose JAT (JATH) and DONE treatment have higher mean OTU numbers and alpha diversities (Figs. 4a,b, S1), indicating that JAT administration can improve the abundance of gut microbiota. The mice fecal microbiota was generally clustered consistently within groups. PCoA and NMDS indicated that microbiota of the mice that received JAT administration were more similar among C57BL/6 than APP/PS1 mice. This finding implies JAT treatment may restore the gut microbiota balance in APP/PS1 mice (Fig. 4c,d). Sample hierarchical clustering using unweighted pair-group method with arithmetic mean (UPGMA) based on OTU or phylogenetic showed that the microbiota of mice feces was different among mouse strains and groups. The microbiota in C57BL/6 mice and group JATH showed greater similarity with closer matrix distances than with samples from other groups (Fig. 4e,f).

#### Abundance of some bacteria is also changed through JAT administration

Distinct taxonomic patterns discriminate APP/PS1 mice from C57BL/6 mice, while high dose JAT and DONE administration blurs the difference (Fig. 5a). JATH, JATL as well as DONE administered APP/PS1 mice had elevated the amount of Phylum *Tenericutes* and *Bacteroidetes* compared to saline treated group (Fig. 5b,c). The most predominant phyla *Firmicutes* was downregulated in JATH (mouse strain) groups compared to APP/PS1 saline group. Low dose JAT administration also down-regulated phyla *Firmicute*, although it was not significant (Fig. 5d). The ratio of Firmicutes and Bacteroidetes (F/B), an important microbiological indicator used to measure obesity, is an independent risk factor for Alzheimer’s disease. Individuals with a high obesity index have greater F/B values. APP/PS1 mice have greater F/B ratio than C57BL/6 mice (p < 0.001). Our results, as shown in Fig. 5e, revealed that JAT treatment can lower the F/B ratio.

*Lactobacillus acidophilus* and *Bifidobacterium* were the main probiotic in intestine, which play crucial roles in the maintenance of gastrointestinal micro-ecological balance^39^. APP/PS1 mice showed less quantities of these two beneficial microbes compared to C57BL/6 mice, while JATH treatment elevated their amount (Fig. 5f,g). *Faecalibaculum* is a putative anti-inflammatory taxon colonizing in intestine^12^, and our data showed that its amount was higher in the JATH treated and C57BL/6 mice compared to the APP/PS1 control group that received saline (Fig. 5h).

#### Functional analysis and co-expression analysis of all mice

**Figure 6:**
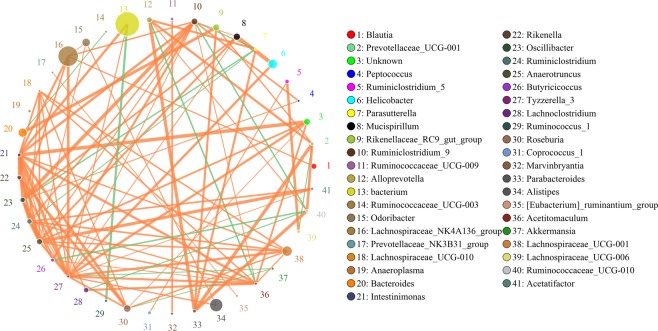
Functional Analysis and co-expression analysis of all mice. SparCC was used for correlation analyses and the image revealed the network diagram of genera with a correlation coefficient greater than 0.1.

The functional genes involved in the metabolic pathway differed significantly between C57BL/6 and APP/PS1 mice. In addition, we also observed that these genes were also altered in mice that were treated with drugs (Fig. S2). SparCC was used for correlation analyses in order to elucidate the complex inner workings of microbial communities according to the abundance and variation of each genus in each sample. Figure 6 shows the network diagram of genera with a correlation coefficient greater than 0.1. In this study, we observed a strong correlation among some genus, within genus *Helicobacter* and *Mucispirillum* show the strongest positive correlation *(r* = *0.886)*, and *Bacterium* and *Ruminococcus_1* had the strongest negative correlation in this study *(r* = −*0.767)*.

## Discussion

AD is the most common neurodegenerative disorder with impaired cognition^9^. It is generally considered that both environmental and genetic factors are involved in AD pathogenesis. Genes associated with amyloid metabolism, tau phosphorylation, lipids transport, cell migration and inflammatory pathways were considered the most susceptible genes in AD^13^. While some non-genetic environmental factors such as exposure to hazardous materials, lifestyle and physical status were thought to be more important than genetic factors to cause AD, increasing evidence suggests that the etiology of AD could also derive from the gut microbiota^40^.

Our previous studies have shown that JAT had significant protective effects against H_2_O_2_-induced injury on nerve cells^23^and against Aβ_25–35_-induced neurotoxicity on rat cortical neurons via its antioxidative potential. Therefore, in light of these findings, we are committed to unraveling the potential therapeutic effect of JAT on AD through *in vivo* experiments. In the current study, at the age of 3 months, the APP/PS1 mice were treated with JAT for 24 weeks, followed by behavioral evaluation using the Morris water maze. In order to assess the effectiveness of JAT in AD, Donepezil hydrochloride was selected as the positive control in behavioral tests. JAT improved the spatial learning and spatial memory abilities, as reflected by acquisition and probe trials of the Morris water maze. However, the mechanisms by which these beneficial effects of JAT are utilizing on Alzheimer’s disease are still enigmatic. Previous research reported that Aβ plaques can be divided into diffuse and dense-core plaques. Differing from dense-core plaques, diffuse plaques are incapable of being stained by ThS due to the lack of a special aggregated β-sheet structure^41^, which is present in dense-core plaques. We performed the immunostaining in the mice brains from each group after undergoing behavioral test. Microscopic observations revealed that JAT treatment significantly reduced Aβ plaques in the cortex and hippocampus of APP/PS1 mice.

Donepezil is a long-acting drug inhibited the activity of acetylcholinesterase^24^, and thus enhances cognitive function. It was widely used as the positive control in AD models^42–44^. Results in this study supported that donepezil treatment ameliorated learning and memory deficits in APP/PS1 transgenic mice, which was consistent with previous research^45^. At the same time, we also observed that donepezil treatment regulated abundance of some bacteria such as *Tenericutes*, *Bacteroidetes* as well as *Firmicutes*. However, as an acetylcholinesterase inhibitor (AChE), donepezil can cause gastrointestinal side effects, such as nausea, vomiting and other diseases^46^, while the recommended doses of JAT seem have fewer side effects. On the other hand, the donepezil treatment reduced the abundance of several OTUs species of Bifidobacterium and Lactobacillus, while JAT treatment increased the corresponding abundance. Bifidobacterium and Lactobacillus are both short-chain fatty acids (SCFAs) -producing strains in the intestinal tract, and various studies have shown that the gut microbiota benefits humans through SCFA production, and the lack of SCFA production is associated with diseases^47^. Therefore, we proposed that, compared with the Jatrorrhizine treatment, donepezil might result in an imbalance of gut microbiota.

To the best of our knowledge, the present study is the first one to evaluate the effect of JAT on gut microbiota in the APP/PS1 mouse model of Alzheimer’s disease. We showed APP/PS1 mice had less gut microbiota compared to C57BL6 mice. The number of OTUs and alpha-diversity were in a significantly lower level in APP/PS1 mice, a finding which was consistent with some other studies on gut microbiota between AD mouse model and normal controls^48^. Furthermore, JAT treatment elevated the alpha diversity and OTU numbers in APP/PS1 mice. In respect of gut microbiota composition, phyla *Bacteroidetes* and *Firmicutes* are dominant divisions of gut flora^49^. In our study, the number of phyla *Bacteroidetes* was decreased while that of phyla *Firmicutes* was more abundant in APP/PS1 mice compared to C57BL/6 mice. Interestingly, we discovered that JAT administration reversed the phenotype. Other reports also showed that amount of *Firmicutes* was increased while *Bacteroidetes* were decreased in the 5xFAD mouse model when compared to non-transgenic 9-week-old wild-type littermates^14^. In another AD mouse model, the proportion of *Bacteroidetes* and *Firmicutes* between APP/PS1 mice and WT are opposite, and showed that the ratio of *Bacteroidetes* to *Firmicutes* increased with age^19^. These findings may indicate that the gut microbiota varies from model to model in AD, and this variation may be related to varying ages of the mice as well. Further investigation is needed to understand the underlying mechanism of the diverse distribution of gut microbiota.

Studies have found that beneficial bacteria can reduce stress and anxiety responses, reduce repetitive behaviors, and improve cognitive function and communication in animals^50^. Interestingly, we found that JAT treatment enriched the amounts of beneficial bacteria, such as *Faecalibaculum, Lactobacillus acidophilus* and *Bifidobacterium*. The fact that JAT treatment elevated the alpha diversity, OTU numbers and gut microbiota composition, indicates that JAT administration can ameliorate microecology dysbiosis in APP/PS1 mice. Taking all data from the previous studies into consideration, the evidence herein strongly suggests that JAT can improve the learning and memory capabilities in APP/PS1 mice possibly by regulating the intestinal flora, in addition to its anti-oxidative stress effects *in vivo*.

Several pharmacological studies indicated that jatrorrhizine has various bioactivities, such as antioxidant, low host toxicity and highly potent antimicrobial activity^51–53^. E-selectin is one of the leukocyte adhesion molecules that can cause endothelial cell dysfunction and increased permeability, which may eventually lead to the dysbiosis of gut microbiome. While in one study^54^, Jatrorrhizine was proved to significantly suppress the inflammatory factor E-selectin as well as TNF-alpha expression secreted by RIMECs, and finally inhibit the the occurrence and development of inflammatory response. Besides, jatrorrhizine has also been shown to reduce blood glucose levels in alloxan diabetic mice and demonstrate acetylcholinesterase inhibitory properties^55^. In addition, jatrorrhizine was reported to regulate the contractions of gastrointestinal smooth muscles by activation of acetylcholine receptors^56,57^. Furthermore, several bacterial species, including Staphylococcus aureus, are capable of producing amyloid fibers^58^, Yu *et al*. studied major single components of Radix Tinosporae, and indicated that alkaloids, such as palmatine and jatrorrhizine, might play main antimicrobial roles on Staphylococcus aureus^59^. Taking these factors into account, JAT might balance the gut microbiota through the above channels. In this case, further research is necessary to fully elucidate the molecular mechanisms.

The gut microbiota-brain axis theory suggests that there is a crosstalk between gastrointestinal microbiota and its human host. Growing evidence has led to the recognition that secretory or degradation products of human microbes play pivotal roles in the etiology of many human diseases. An interesting study carried out by Uemura N and his coworkers provide direct evidence to show that Parkinson pathogens, different forms of α-synuclein, if inoculated into the mouse gastrointestinal tract, could spread from the gastrointestinal tract to the brain via the vagal nerve in rats^60^. A clinical matched-cohort study also implied a potential protective effect of truncal vagotomy against the development of Parkinson Disease^61^. It is suggested vagus nerve maybe a direct channel for substance transporting between brain and gut. It has been reported that vagus activity affects neurogenesis and brain-derived neurotrophic factor (BDNF) mRNA expression in adult hippocampus^62^. Research has showed that one of the primary neurotransmitters used by the vagus nerve is acetylcholine^63^ and the vagal innervation may modulate the intestinal microenvironment by determining the cholinergic tone in the enteric nervous system^64^. Coincidentally, one more study reported the enteric nervous system can amplify the vagus nerve to induce a substantial release of ACh in the intestinal microenvironment to regulate the immune response^64,65^. Crumeyrolle-Arias M *et al*. showed that bidirectional interaction between the gut–brain axis and the microbiota promotes communication between the humoral, neurological, immune and endocrine systems^66^. AD mouse showed lower gut microbiota diversities and gut microbiota was reported to regulate host immunity and affect Aβ amyloidosis^19^. However, much remains to be illuminated about the mechanisms and effects of the interaction between the nervous and gastrointestinal systems.

Generally, bacterial interventions in the intestine, including oral microbiome, fecal microbiota transplantation and antibiotic or probiotic administration are often used to explore the effect of gut microbiota on host’s behavior and brain physiology. For instance, in one study, *Enterobacteria* infection exacerbated progression of AD by promoting immune hemocyte recruitment to the brain in an AD Drosophila model^67^. Germ-free APP/PS1 transgenic mice exhibited reduced Aβ deposition in brain compared to specific pathogen free mice, while colonization of Germ-free APP/PS1 mice with microbiota increases Aβ pathology^19^. Probiotic administration on 3xTg-AD mice modifies intestinal microbiota and counteracts cognitive decline and brain damage^68^. These *in vivo* microbiota reconstruction experiments provide new insights into further prevention and treatment of AD.

## Conclusion

In summary, the current study demonstrated that APP/PS1 mice display gut dysbiosis, treatment with JAT improved hippocampal contextual memory and altered the abundance of some specific gut microbiota such as the most predominant phylum Firmicutes, Bacteroidetes and Lactobacillus as well as F/B ratio in APP/PS1 mice. This may provide a therapeutic way to balance the gut dysbiosis in AD patients.

## Supplementary information

The JAT treatment modulated the imbalance of gut microbiota in APP/PS1 transgenic mice.

## Data Availability

16S rDNA amplicon sequencing data were submitted to the NCBI SRA database, accession number PRJNA590832. The datasets supporting the conclusions of this article are included within the article.
