# Supplementary material for: Jatrorrhizine Balances the Gut Microbiota and Reverses Learning and Memory Deficits in APP/PS1 transgenic mice
Source: Sci Rep. 2019 Dec 20;9:19575. doi: 10.1038/s41598-019-56149-9 (PMC6925119; doi:10.1038/s41598-019-56149-9)
Supplement: Supplementary file 1 — The JAT treatment modulated the imbalance of gut microbiota in APP/PS1 transgenic mice. [file 41598_2019_56149_MOESM1_ESM.pdf]

Title

Jatrorrhizine Balances the Gut Microbiota and Reverses Learning and Memory Deficits in APP/PS1 transgenic mice

Author Lists

Sheng Wang[#1],Wei Jiang[#2], Ting Ouyang[1], Xiu-Yin Shen[1], Fen Wang[1],

Yu-hua Qu[3]， Min Zhang[1], Tao Luo[1]*, Hua-Qiao Wang[1]*

1. Department of Anatomy and Neurobiology, Zhongshan School of Medicine, Sun Yat-sen University, Guangzhou, Guangdong, 510080, China.

2. Department of Anatomy, Histology and Developmental Biology, School of Basic Medical Sciences, Shenzhen University Health Science Centre, Shenzhen 518060, China

3. Pediatric Hematology and Oncology, Affiliated Guangzhou Women and Children's Hospital, Zhongshan School of Medicine, Sun Yat-Sen University, Guangzhou 510623, China

[#] These authors contribute equally to the work.

***Address correspondence to these authors at:** (Hua-Qiao Wang) Department of Anatomy and Neurobiology, Zhongshan School of Medicine, Sun Yat-sen University, Guangzhou, Guangdong, 510080, China; Tel: +8602087332218; Fax: +86 02087330709; E-mail: wanghq@mail.sysu.edu.cn and (Tao Luo) Department of Anatomy and Neurobiology, Zhongshan School of Medicine, Sun Yat-sen University, Guangzhou, Guangdong, 510080, China; Tel: +8602087332218; Fax: +86 02087330709; E-mail: luot8@mail.sysu.edu.cn

Author Lists:

Sheng Wang wangsh273@mail2.sysu.edu.cn

Wei Jiang jiangwei@szu.edu.cn

Ting Ouyang 523704243@qq.com

Xiu-Yin Shen 460188162@qq.com

Fen Wang 1291147006@qq.com

Yu-hua Qu quyuhua2007@163.com

Min Zhang zforensicm@163.com

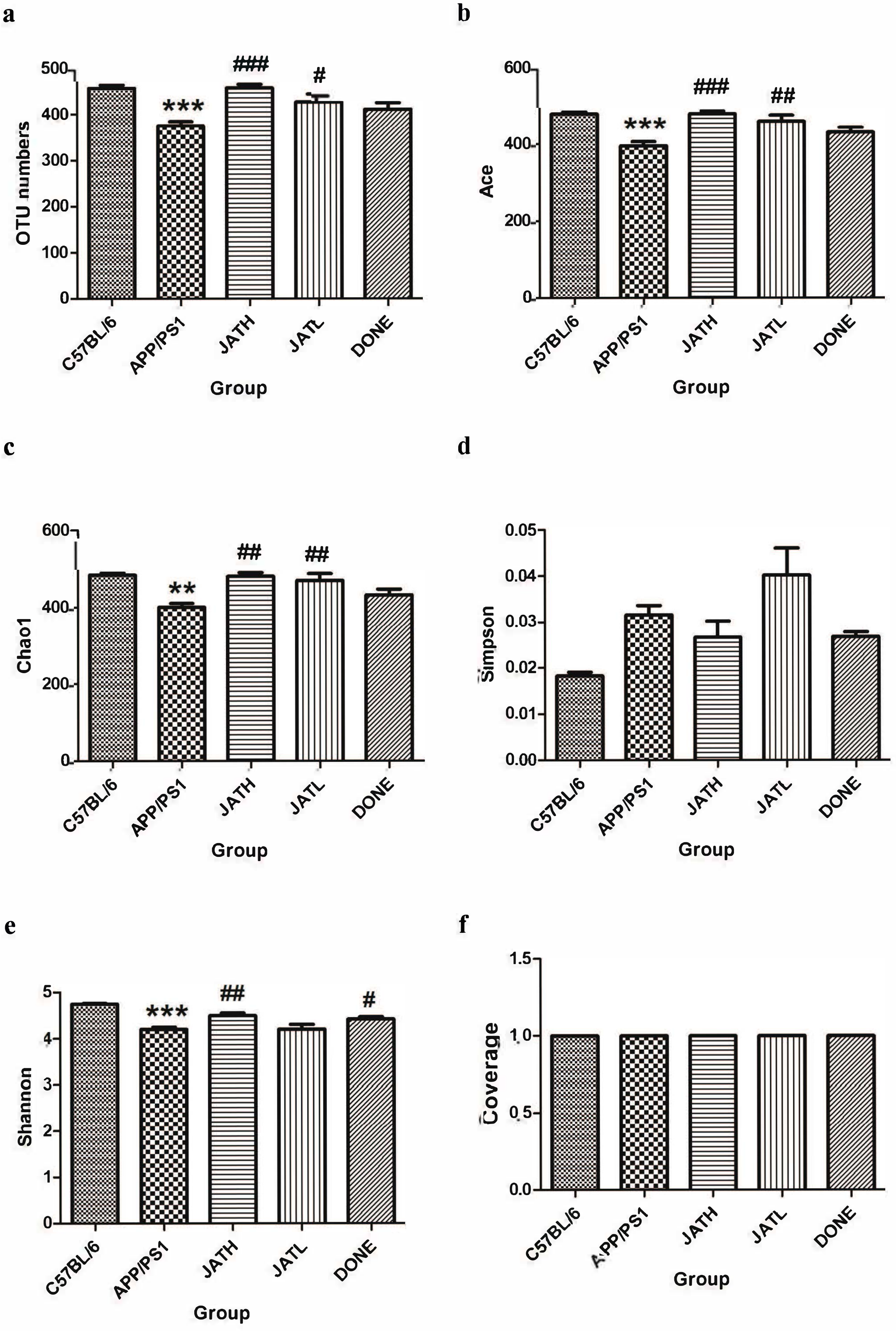

Figure Supplement 1

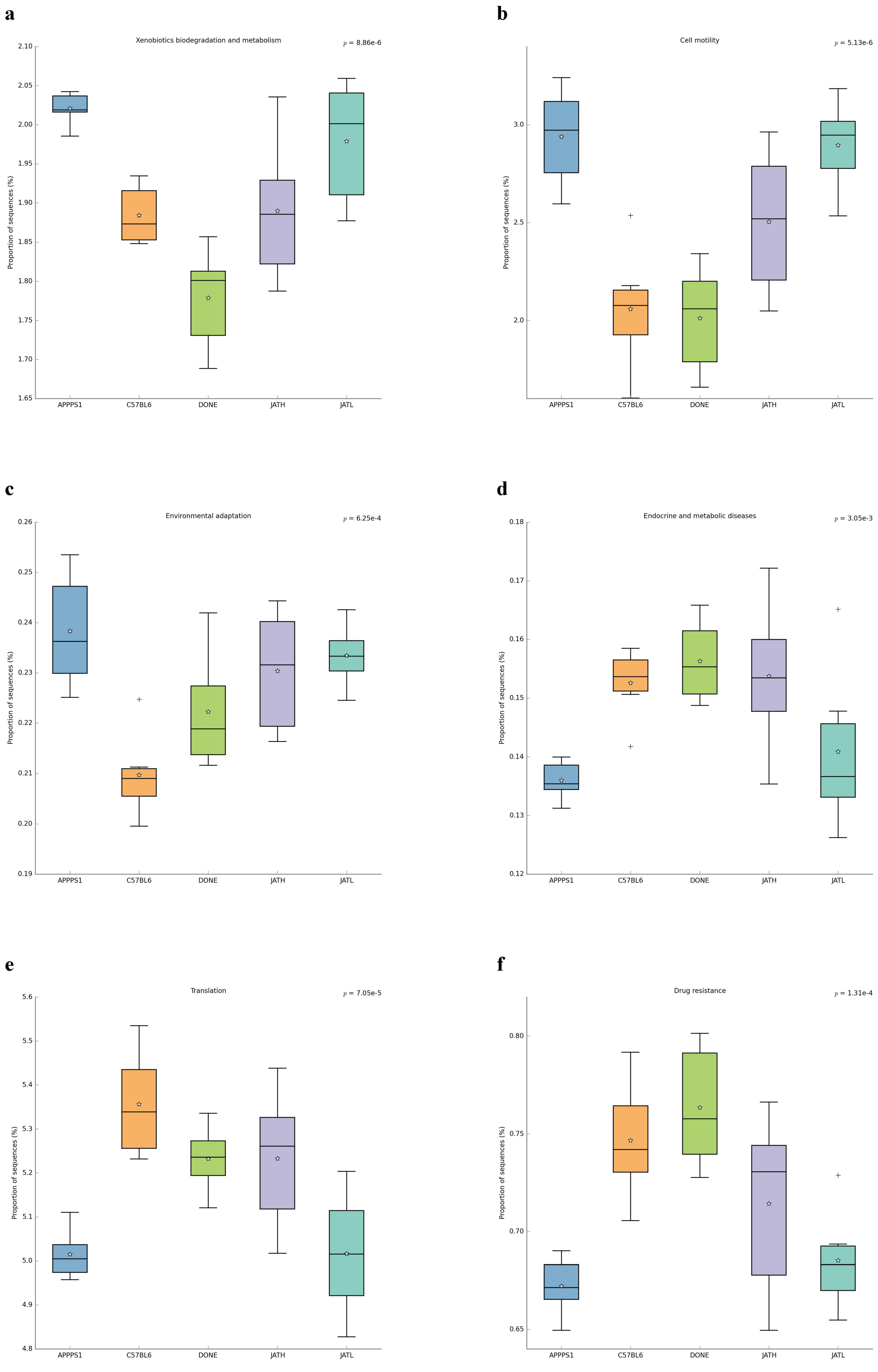

Figure Supplement 2
